# Myeloid Derived Suppressor Cells Are Present at High Frequency in Neonates and Suppress *In Vitro* T Cell Responses

**DOI:** 10.1371/journal.pone.0107816

**Published:** 2014-09-23

**Authors:** Ana Gervassi, Nicholas Lejarcegui, Sandra Dross, Amanda Jacobson, Grace Itaya, Elvis Kidzeru, Soren Gantt, Heather Jaspan, Helen Horton

**Affiliations:** 1 Seattle Biomedical Research Institute, Seattle, Washington, United States of America; 2 University of Washington Department of Global Health, Seattle, Washington, United States of America; 3 University of Washington Department of Medicine, Seattle, Washington, United States of America; 4 University of Washington Seattle Children's Hospital, Seattle, Washington, United States of America; 5 Division of Immunology, Institute of Infectious Diseases and Molecular Medicine, University of Cape Town, Cape Town, South Africa; 6 University of British Columbia Department of Pediatrics and Child and Family Research Institute, Vancouver, Canada; UNIFESP Federal University of São Paulo, Brazil

## Abstract

Over 4 million infants die each year from infections, many of which are vaccine-preventable. Young infants respond relatively poorly to many infections and vaccines, but the basis of reduced immunity in infants is ill defined. We sought to investigate whether myeloid-derived suppressor cells (MDSC) represent one potential impediment to protective immunity in early life, which may help inform strategies for effective vaccination prior to pathogen exposure. We enrolled healthy neonates and children in the first 2 years of life along with healthy adult controls to examine the frequency and function of MDSC, a cell population able to potently suppress T cell responses. We found that MDSC, which are rarely seen in healthy adults, are present in high numbers in neonates and their frequency rapidly decreases during the first months of life. We determined that these neonatal MDSC are of granulocytic origin (G-MDSC), and suppress both CD4+ and CD8+ T cell proliferative responses in a contact-dependent manner and gamma interferon production. Understanding the role G-MDSC play in infant immunity could improve vaccine responsiveness in newborns and reduce mortality due to early-life infections.

## Introduction

Despite progress in reducing the infant mortality rates over the last two decades, infectious disease remains a major cause of infant mortality, with an estimated 4.9 million deaths per annum [1]. A major goal of neonatal vaccinology is the induction of protective immunity before the age at which most infections occur. Development of vaccines that can induce protective immunity at this vulnerable age has been hampered in part by differences in T cell responses during infancy [2]–[5]. The neonatal immune system is biased to tolerogenic and Th2 type responses, compared to older children and adults [6]. We hypothesized that one reason for altered T cell responses in early life may be active suppression by myeloid-derived suppressor cells (MDSC), a heterogeneous population of activated myeloid cells with suppressive function [7]–[9] [10]. While a tolerant, anti-inflammatory state is likely advantageous for full-term viviparity [11]–[13], its persistence after birth may contribute to the reduced ability of infants to respond to infections and vaccinations in early life.

In certain pathologies, in particular cancer and persistent inflammatory conditions, an accumulation and activation of granulocytic or monocytic MDSC that express suppressive factors such as Arginase-1, reactive oxygen species, and inducible nitric oxide synthase [7]–[9] has been observed. The vast majority of research on MDSC to date has focused on populations of MDSC induced in murine cancer models and in humans with malignancy [7]–[9]. Recently, high frequencies of granulocytic (G)-MDSC were described in cord blood [14]. In this study, we confirm these findings and further characterize the frequency and immunosuppressive function of this G-MDSC population.

G-MDSC express cell markers similar to neutrophils and recently, mature neutrophils have been found to be either inflammatory (N1) or immunosuppressive (N2) [15]–[17]. The relationship between the mature immunosuppressive neutrophils and G-MDSC has not been established, however murine transcriptomic analysis has revealed significant differences between G-MDSC and suppressive mature neutrophils [18]. We therefore also examined the nuclear morphology and heterogeneity of the population of G-MDSC further differentiating them from mature neutrophils.

## Materials and Methods

### Sample collection and processing

Adult blood samples were collected from healthy volunteers at the Seattle Biomedical Research Institute. Cord blood from healthy, full-term Caesarean section deliveries was collected at the Valley Medical Center, Department of Obstetrics and Gynecology, University of Washington (UW). Blood from healthy 6-week old babies was collected during study visits at the Khayelitsha Day Hospital, Provincial Administration of the Western Cape, University of Cape Town. Blood from 6–24 month-old healthy infants was collected during elective surgeries at Seattle Children's or UW. The Institutional Review Boards from Seattle Biomedical Research Institute, UW, Valley Medical Center and University of Cape Town approved the studies and all adult individuals provided written informed consent and guardians provided proxy consent for infants.

Cord blood mononuclear cells (CBMC), infant, or adult PBMC were isolated over Ficoll-Hypaque gradients. CBMC were further depleted of red blood cells by glycophorin A negative selection (Miltenyii Biotech). All assays were performed within 8 hours of collection of cord blood or peripheral blood since G-MDSC do not survive cryopreservation (data not shown and [19]).

### Phenotypic analysis of G-MDSC populations and flow cytometry

Antibodies against the following surface antigens were used to identify G-MDSC populations: HLA-DR (L243), CD14 (M5E2), CD11b (ICRF44), CD33 (WM53), purchased from BD Biosciences, and CD15 (HI98, BioLegend). Intracellular staining was detected after permeabilization and staining with previously labeled Alexa Flour 488 (Invitrogen) anti-arginase-1 antibody (clone 6G3; Hycult Biotechnologies). Viable cells were identified by staining with Live Dead Amine (Invitrogen). All stained samples were fixed in 1% paraformaldehyde and acquired using an LSRII Flow cytometer (BD). Data were analyzed using FlowJo software (Tree Star).

### Neutrophil, G-MDSC and T cell enrichment

G-MDSC and T cells were isolated from the CBMC interface of a Ficoll gradient by CD15 (Miltenyii Biotech) or CD3 (EasySep) positive magnetic bead selection, respectively. For the isolation of naïve adult T cells, T cells were enriched by negative selection (EasySep) followed by depletion of memory T cells using CD45RO magnetic beads (Miltenii Biotech). Neutrophils were isolated from the red blood cell (RBC) pellet of a Ficoll gradient by Ammonium Chloride RBC lysis (EasySep) followed by neutrophil enrichment (EasySep). Purity of the positively and negatively selected subsets was greater than 95%.

### Wright-Giemsa Stain

Purified neutrophils and G-MDSC were processed for cytospin and Wright-Giemsa stains and analyzed at the Clinical Pathology Laboratory at Seattle Children's Hospital.

### T cell suppression assay

Effector CBMC or CD3^pos^ T cells were CFSE-labeled (Molecular Probes) and cultured at 2×10^6^ cells/ml. T cells were stimulated with anti-CD3/CD28 coated beads (BD) in RPMI plus 15% human sera with the daily addition of 0.1 ng/mL rhuIL-7 (R&D Systems). CD15^pos^ MDSC were depleted from CBMC or added to purified T cell cultures at a 1∶1 effector-to-suppressor ratio. Negative controls included effector cells with or without suppressor cells, without anti-CD3/CD28 stimulation. On day 5, cells were permeabilized, stained with anti-CD3 (UCHT1 or SK7), CD4 (RPA-T4), CD8 (SK1) from BD Biosciences and proliferation of T cells was assessed by analysis of lymphoid-gated, CD3^pos^, CFSE^lo^ cell populations. Positive proliferative responses were calculated after subtraction of background proliferation from corresponding negative control wells. Percent suppression was calculated by the following formula: %CFSE^lo^ (CBMC) - %CFSE^lo^ (CBMC-CD15)/%CFSE^lo^ (CBMC-CD15) ×100.

### IFN-gamma ELISpot analysis

Effector CBMC or CD15-depleted CBMC were plated at 100,000–200,000 PBMC or 20,000 purified CD3^pos^ T cells per well in IFN-γ ELISPOT assays (Millipore), as previously described [20], [21] with the addition of IL-12 (10 ng/mL) and IL-7 (1 ng/mL, R&D Systems) for the neonatal T cells. The number of spot forming cells (SFC) was calculated by subtracting the mean number of spots in the negative control wells from the mean number of spots for each stimulation condition. An IFN-γ result was considered positive when the background-subtracted number of SFC was twice the background (negative control) and at least 50 SFC per million CBMC or T cells.

### Statistical analysis

For statistical analysis of G-MDSC frequency comparisons by age group and T cell proliferative responses in the presence or absence of G-MDSC, Mann-Whitney and the Wilcoxon Matched-Pair Signed Rank tests were used, respectively. The correlation between G-MDSC frequencies and suppression of T cell proliferation by G-MDSC was measured using the Spearman rank correlation test. Non-parametric tests were two-tailed, with a *P* value of less than 0.05 considered statistically significant. All statistical analyses and graphing were conducted using GraphPad Prism 5.0 d (GraphPad).

## Results

### G-MDSC are present at high frequency at birth and gradually decline with postnatal age

**Figure 1:**
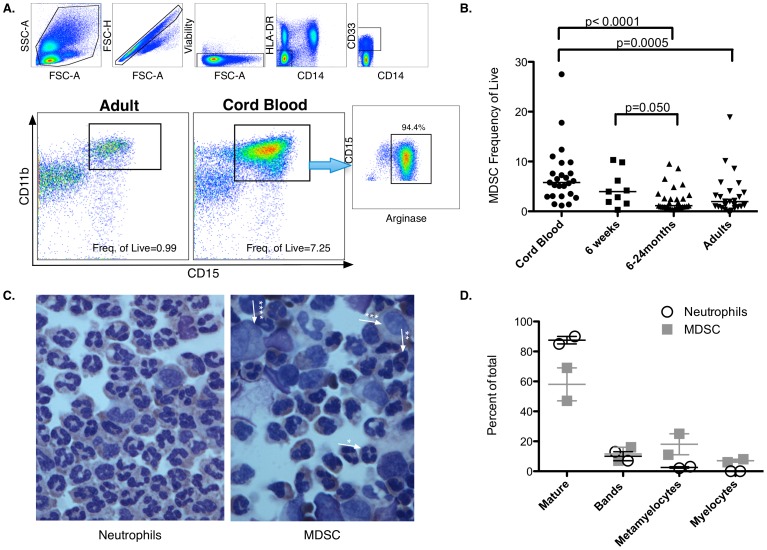
Characterization and longitudinal analysis of MDSC populations in cord blood, infants and adults. (**A**) Gating strategy and identification of HLA-DR/CD14^neg^, CD33/CD11b/CD15^pos^ G-MDSC in adult and cord blood. Further characterization of HLA-DR/CD14^neg^, CD33/CD11b/CD15^pos^ cells by intracellular staining of Arginase I. (**B**) Frequency of HLA-DR/CD14^neg^, CD33/CD11b/CD15^pos^ cells of: (i) CBMC isolated from CB collected from healthy pregnancies in Seattle, WA (n = 25); (ii) PBMC isolated from neonates in Cape Town, South Africa at 6-weeks of age (n = 9); (iii) PBMC isolated from 6–24 month-old infants in Seattle, WA (n = 29); (iv) and PBMC isolated from healthy adults in Seattle, WA (n = 28). Statistical significance determined by the Mann Whitney test. (**C**) Wright-Giemsa cytospin of CB samples and phenotype determination by clinical pathology of neutrophils and G-MDSC (Average, n = 2 independent experiments). Magnification 600X. (**D**) Proportions of neutrophils at various stages of development in the neutrophil and the G-MDSC fractions.

To establish the prevalence of MDSC during early life, MDSC frequencies were analyzed by flow cytometry in blood mononuclear cells isolated from healthy (i) cord blood (CB); (ii) 6-week-old infants; (iii) 6–24 month-old infants; and (iv) adults. Frequencies of both monocytic MDSC (M-MDSC; defined as HLA-DR^neg^, CD14^pos^, CD33^pos^, CD11b^pos^) and G-MDSC (defined as HLA-DR^neg^ CD14^neg^, CD33^pos^, CD11b^pos^, and CD15^pos^) were quantified (Fig.1A and Fig. S1 for fluorescence minus one (FMO) controls). The frequencies of M-MDSC were below 1% of live cells, did not differ significantly between cord blood and adult peripheral blood, and were, therefore, not analyzed further. We identified a prominent population of G-MDSC in CB that decreased with age and were generally absent in healthy adults (median frequencies: 5.8% in CB; 4% at 6-weeks of age; 1.2% between 6–24 months and 0.8% in adults, Fig.1B). Over 90% of these cells were positive for Arginase-1 (Fig.1A), confirming their granulocytic nature [22]–[25]. Interestingly, G-MDSC frequencies observed in CB were typical of those reported in peripheral blood of cancer patients [23]–[25]. CB G-MDSC were further characterized by Wright Giemsa stain, which showed heterogeneous populations of hypodense-immature and mature neutrophils (47–69% neutrophils; 7–16% bands; 11–25% metamyelocytes and 6–8% myelocytes, based on 2 experiments; Fig.1C,D) further confirming these as G-MDSC.

### Isolated neonatal T cells have more variable proliferative responses compared to adult naïve T cells in response to polyclonal stimulation

**Figure 2:**
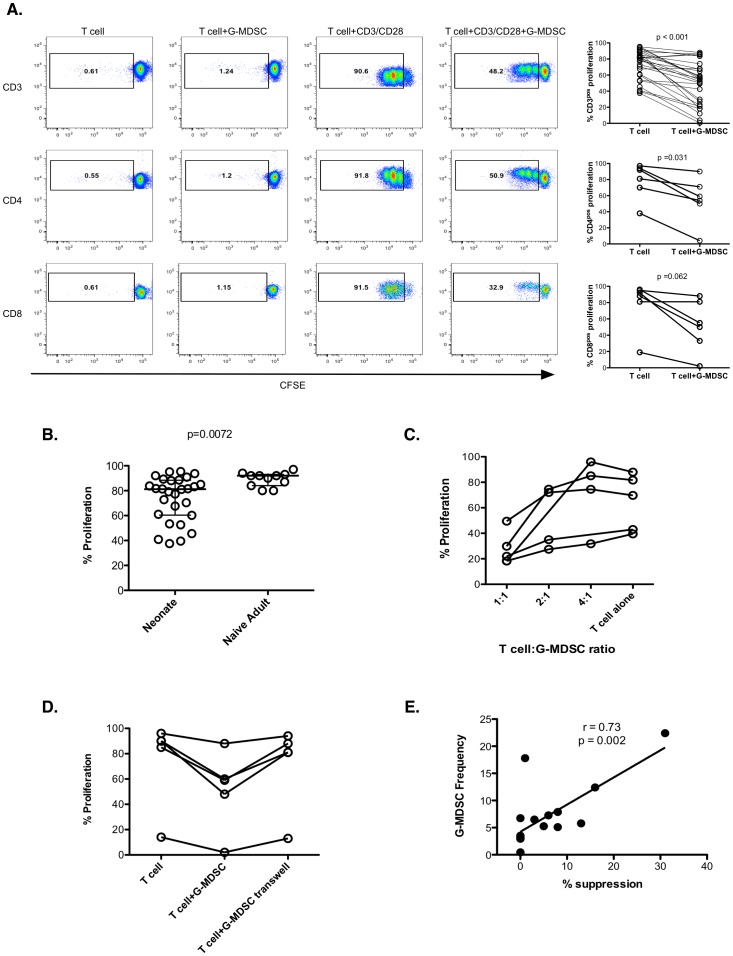
Effect of neonatal G-MDSC on T cell proliferation. (**A**) Proliferative responses of purified T cells in the presence or absence of G-MDSC after anti-CD3/CD28 bead stimulation (n = 28 independent experiments performed in duplicate for CD3 plots, n = 6 for CD4 and CD8 plots). Significance determined by the Wilcoxon Matched-Pair Signed Rank test. (**B**) Proliferative responses of purified adult naïve T cells (n = 9 independent experiments performed in duplicate) compared to cord blood T cells (n = 28 independent experiments performed in duplicate) after anti-CD3/CD28 bead stimulation. Statistical significance determined by the Mann Whitney test. (**C**) Suppression of T cell proliferation by autologous G-MDSC titration. (n = 4 independent experiments performed in duplicate). (**D**) Suppression of T cell proliferative responses by G-MDSC is contact dependent. (n = 5 independent experiments performed in duplicate). (**E**) G-MDSC frequency correlates with suppression of T cell proliferation by G-MDSC. G-MDSC frequencies were correlated to suppression of T cell proliferation by G-MDSC using the Spearman rank correlation test (n = 16 independent experiments).

Neonatal T cell responses to infection and vaccines are reduced compared to adults with a delay in the acquisition of antigen-specific CD4^pos^ T cell responses as well as decreased cytokine responses [6]. We therefore sought to assess if neonatal T cells have a decreased ability to proliferate in response to polyclonal stimulation when isolated from the rest of cord blood peripheral mononuclear cells (CBMC). To assess this, T cells were purified using magnetic beads and stimulated with anti-CD3/anti-CD28 beads. Adult T cells were further enriched for naïve T cells (CD45RO^neg^). The proliferative capacity of T cells was measured by CFSE dilution five days after stimulation (Fig. 2A). As shown in Fig. 2B, neonatal T cells show highly variable proliferative capacity compared to naïve adult T cells, with a lower median proliferation (p = 0.008).

### Neonatal G-MDSC further inhibit T cell proliferative responses *in vitro* in a contact-dependent manner

In cancer patients, increased G-MDSC frequencies have been recognized as potent inhibitors of T cell responses, limiting the effectiveness of immunotherapy [23]–[25]. In order to assess whether infant G-MDSC behave similarly, T cells and G-MDSC were purified from CBMC using anti-CD3 or anti-CD15 magnetic beads, respectively. CFSE-labeled CB-derived CD3^pos^ T cells were stimulated with anti-CD3/anti-CD28 beads with or without G-MDSC (Fig. 2A). Addition of G-MDSC resulted in significantly reduced proliferative capacity of neonatal T cells (Fig. 2A; p<0.001). This reduction in proliferative capacity induced by G-MDSC occurred in both CD4^pos^ and CD8^pos^ T cells (Fig. 2A). Furthermore, the suppressive effects of G-MDSC on neonatal T cell proliferation showed a dose response (Fig. 2C). Finally, in order to assess if the effect of G-MDSC is contact dependent, T cells and G-MDSC were separated by a 0.45 um transwell (Fig. 2D). Separating G-MDSC from T cells with the transwell eliminated the suppressive ability of G-MDSC, indicating that their effect is entirely contact dependent.

### The frequency of neonatal G-MDSC correlates with the proliferative capacity of CBMC after polyclonal stimulation

To analyze the effect of G-MDSC on the capacity of T cells in whole CBMC (rather than isolated T cells) to proliferate after polyclonal stimulation, G-MDSC were depleted by CD15 bead isolation from CBMC. CFSE-labeled CBMC or CD15-depleted CBMC were stimulated with anti-CD3/anti-CD28 beads. As shown in Fig. 2E, the amount of T cell suppression directly and significantly correlated with the frequency of G-MDSC in CB (r = 0.73, p = 0.002).

### Neonatal T cells produce lower amounts of IFN-gamma compared to naïve adult T cells, this is further suppressed by neonatal G-MDSC

**Figure 3:**
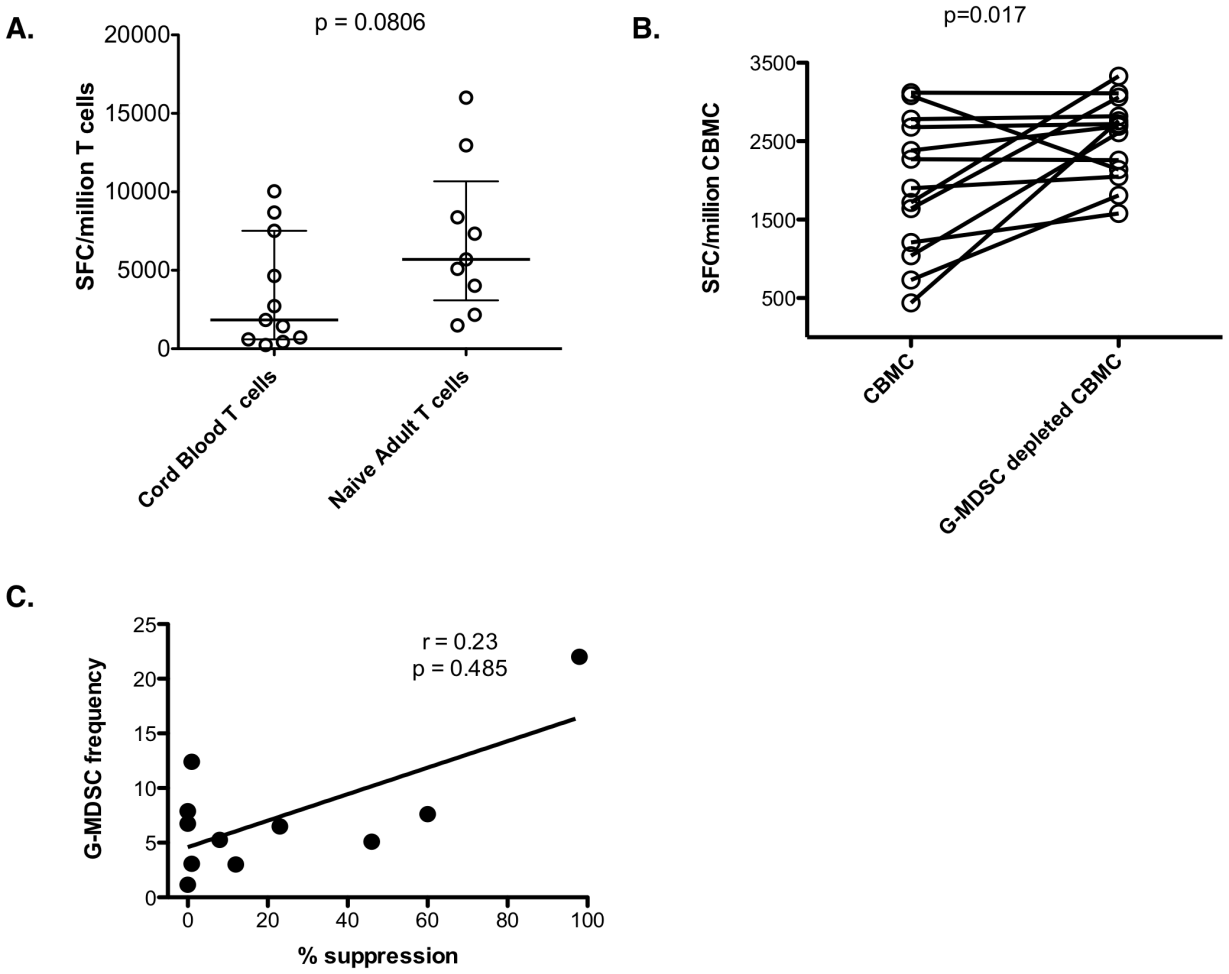
Effect of G-MDSC on IFN-gamma production. (**A**) Cord blood CD3^pos^ T cells and adult CD3^pos^ CD45RO^neg^ T cells were assessed for IFN-gamma production by ELISpot after anti-CD3/CD28 bead stimulation (n = 11 independent experiments performed in triplicate for neonates and 9 independent experiments for adults). Statistical significance determined by the Mann Whitney test. (**B**) Neonatal G-MDSC decrease IFN-gamma production after anti-CD3/CD28 bead stimulation. (n = 13 independent experiments performed in triplicate). (**C**) G-MDSC frequency correlation with suppression T cell of IFN-gamma production by G-MDSC. G-MDSC frequencies were correlated to suppression using the Spearman rank correlation test (n = 11 independent experiments).

In order to assess if neonatal T cells are also defective at producing IFN-gamma, CD3^pos^ cells from CB were stimulated with anti-CD3/anti-CD28 beads and compared to naïve CD3^pos^ T cells from adults. As shown in Fig 3A, neonatal T cells have a lower median IFN-gamma secretion though it did not reach significance (p = 0.081).

We sought to assess if neonatal G-MDSC also affect IFN-gamma secretion after polyclonal stimulation since IFN-gamma production is decreased by MDSC in cancer patients [26]. G-MDSC were depleted from whole CBMC by anti-CD15 bead isolation and IFN-gamma responses were compared to non-depleted CBMC by ELISpot after anti-CD3/anti-CD28 stimulation. As shown in Fig. 3B, depletion of G-MDSC significantly increased IFN-gamma production (p = 0.017). However, unlike with the proliferative assays, the frequency of G-MDSC in CB did not correlate with the ability of G-MDSC to suppress IFN-gamma production Fig. 3C, r = 0.23, p = 0.485).

## Discussion

Little is known about what governs the immunologic differences seen in early life, or how these change over time. An immune-suppressive feto-maternal environment appears necessary for healthy full-term gestation, as inflammation has been shown to be associated with pre-term parturition and fetal injury [27]. Several maternal immune-suppressive mechanisms have already been identified, including regulatory T cells, regulatory NK cells, and regulatory molecule expression such as galectin-1, PDL1, and Tim3 [12], [28]–[34], and failure of some of these mechanisms is associated with spontaneous abortion. Active suppression of fetal immune responses *in utero* is also likely necessary because a bidirectional transfer of nucleated cells occurs across the placental barrier and these could initiate an anti-maternal response [35], [36]. A high proportion of regulatory T cells have been described in the fetus, which decrease during gestation and reach adult-levels by term gestation [35], [37]. A second suppressive cell of erythroid origin has recently been identified in CB [38]. Here we have described another population of suppressive cells in early life: a high proportion of G-MDSC in CB of healthy neonates that gradually declines in frequency during the first 6 months of life.

The data presented in this study corroborate and broaden the findings of Rieber et al. [14]. We have confirmed G-MDSC, but not M-MDSC, are present at elevated frequencies in CB and further characterized these frequencies in the first 2 years of life. To this end we demonstrated that by 6 weeks of age, median G-MDSC frequencies decreased by 30% and that median G-MDSC frequencies did not reach adult levels until after 6 months of age. We also confirmed the findings of Rieber *et al.* demonstrating that neonatal G-MDSC suppress CD4^pos^ and CD8^pos^ proliferative and IFN-gamma production responses. While Rieber et al. found that the effect of MDSCs on T or NK cells was partially contact dependent, we found that suppression of T cell proliferative responses by G-MDSC was completely contact dependent [14]. Furthermore, we demonstrated that in the presence of physiological levels of G-MDSC there is a direct and significant correlation between the G-MDSC frequency and the degree of suppression of proliferative responses observed. Lastly, we have characterized the nuclear morphology and composition of this G-MDSC population in neonates compared to mature neutrophils and have demonstrated that G-MDSC isolated from CB contain a heterogeneous mixture of both hypodense mature and immature neutrophils.

Consistent with previous reports [6], we have demonstrated that T cells in neonates are less responsive compared to naïve T cell responses from adults. Isolated neonatal T cells have a lower proliferative capacity and secrete lower amounts of IFN-gamma after polyclonal stimulation *in vitro*. *In vivo*, the ability of neonatal T cells to mount effective responses is likely influenced by the lower expression of B7 family molecules on antigen-presenting cells (APCs) [39] and the defective cytokine production by these cells [40]. T cell responses induced by several routine vaccines are less polyfunctional, less proliferative and produce lower IFN-gamma in infants compared to adults [41]–[47]. The combination of lower capacity of T cells to proliferate and secrete IFN-gamma combined with the increased frequencies of G-MDSC during the first 6 months of life may impair the induction of protective pathogen-specific and vaccine- induced T cell immune responses *in vivo*. The age at which adult-like responses are achieved varies according to vaccine type, but is generally attained between the ages of 6 months to 1 year, which coincides with the age at which we observe G-MDSC levels to decrease. However, additional studies are required to determine whether this temporal association indicates a causal relationship between G-MDSC frequency and vaccine responsiveness in infants.

Neonatal G-MDSC have strong T cell suppressive activity *in vitro* and their frequency in CB correlates with the proliferative capacity of CB T cells in response to polyclonal stimulation *in vitro*. Although the role these cells may play *in vivo* has not yet been defined, MDSC have been implicated in skewing T helper responses towards Th-2 phenotype, impairing NK responses, and inhibiting dendritic cell function [7]–[9], all of which are characteristics described in the neonate's immune dysfunction [2], [4], [48]. In cancer models, G-MDSCs can be differentiated from immunosuppressive to immunogenic TNF-alpha secreting neutrophils after intra-tumoral injection of attenuated *Salmonella* vaccine [49], and therapeutic vaccination to cancer antigens is restored after *in vivo* depletion of MDSC [50]–[56]. Furthermore, in cancer patients, MDSCs can be induced to differentiate and T cell function and vaccine responses have been restored by administration of either Vitamin A or Vitamin D3 [22], [57]–[59]. Vitamin A and Vitamin D supplementation has also been shown to increase protection after vaccination [60], [61]. This raises the intriguing possibility that if MDSC indeed modulate early life immunity, interventions targeting MDSC might be designed that enhance vaccine responsiveness and reduce infectious mortality.

## Supporting Information

Figure S1 **Fluorescence minus one (FMO) control to identify positivity gates for MDSC populations with the following antibody combination: Live Dead Amine, HLA-DR, CD14, CD11b, CD33, CD15, and intracellular staining of Arginase-1.**
(TIFF)
