# Supplementary figures and images for: Myeloid Derived Suppressor Cells Are Present at High Frequency in Neonates and Suppress In Vitro T Cell Responses
Source: PLoS One. 2014 Sep 23;9(9):e107816. doi: 10.1371/journal.pone.0107816 (PMC4172591; doi:10.1371/journal.pone.0107816)

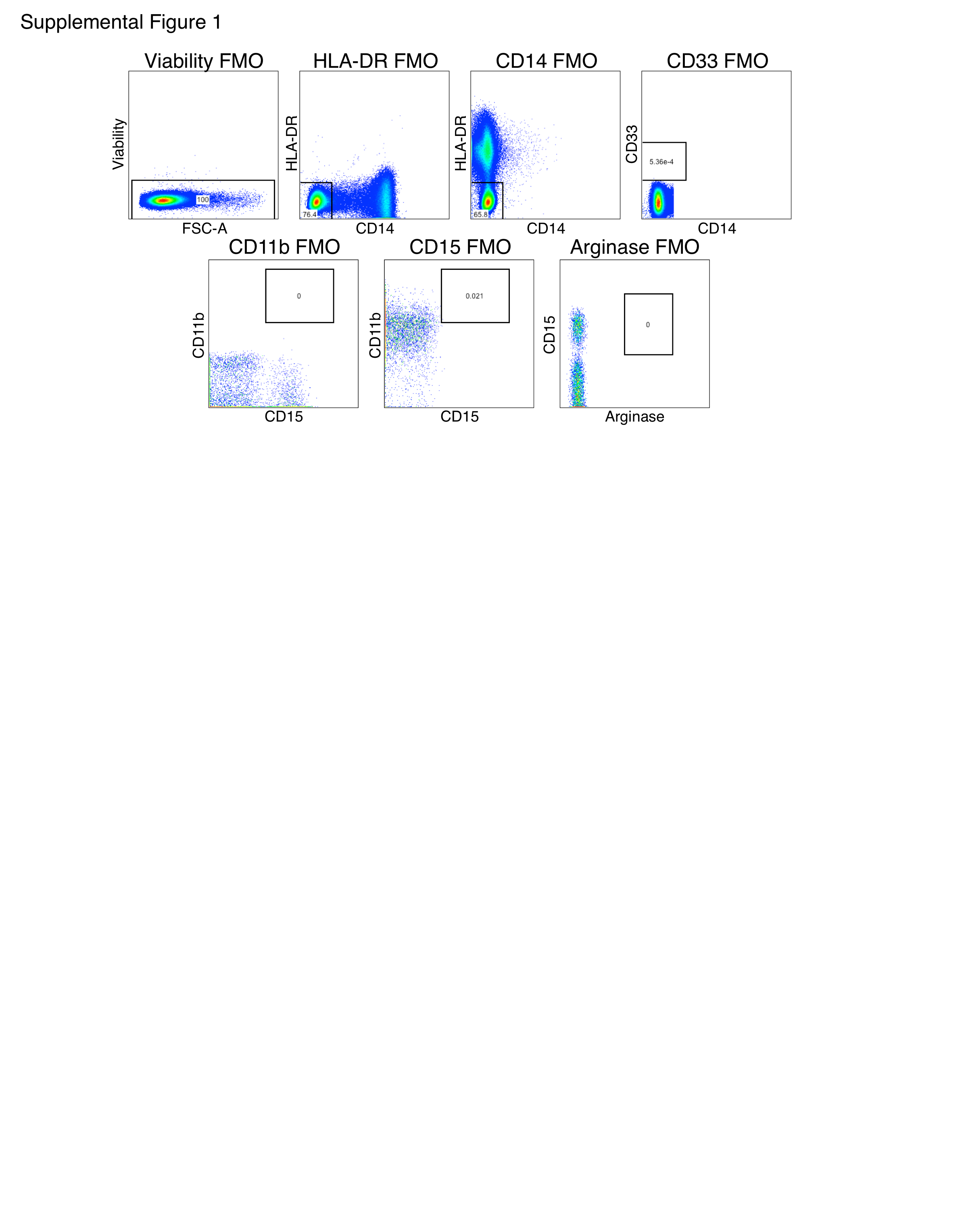

Supplement: Figure S1 — Fluorescence minus one (FMO) control to identify positivity gates for MDSC populations with the following antibody combination: Live Dead Amine, HLA-DR, CD14, CD11b, CD33, CD15, and intracellular staining of Arginase-1. (TIFF) [file pone.0107816.s001.tiff]
